# Influence of Dietary Lipids and Environmental Salinity on the n-3 Long-Chain Polyunsaturated Fatty Acids Biosynthesis Capacity of the Marine Teleost *Solea senegalensis*

**DOI:** 10.3390/md19050254

**Published:** 2021-04-29

**Authors:** Manuel Marrero, Óscar Monroig, Mónica Betancor, Marcelino Herrera, José A. Pérez, Diego Garrido, Ana Galindo, Inmaculada Giráldez, Covadonga Rodríguez

**Affiliations:** 1 Departamento de Biología Animal, Edafología y Geología, Universidad de La Laguna, 38200 San Cristóbal de La Laguna, Spain; janperez@ull.edu.es (J.A.P.); diegogarridolorenzo@gmail.com (D.G.); agg1994@gmail.com (A.G.); covarodr@ull.edu.es (C.R.); 2 Instituto de Acuicultura Torre de la Sal, Consejo Superior de Investigaciones Científicas (IATS-CSIC), 12595 Ribera de Cabanes, Spain; oscar.monroig@csic.es; 3 Institute of Aquaculture, Faculty of Natural Sciences, University of Stirling, Stirling FK9 4LA, UK; m.b.betancor@stir.ac.uk; 4 IFAPA Centro Agua del Pino, 21450 Cartaya, Spain; marcelino.herrera@juntadeandalucia.es; 5 Departamento de Química “Profesor Carlos Vílchez Martín”, Universidad de Huelva, 21007 Huelva, Spain; giraldez@uhu.es

**Keywords:** *Solea senegalensis*, Fads2, Elovl5, LC-PUFA biosynthesis, diet, salinity, EPA, DHA

## Abstract

Fish vary in their ability to biosynthesise long-chain polyunsaturated fatty acids (LC-PUFA) depending upon the complement and function of key enzymes commonly known as fatty acyl desaturases and elongases. It has been reported in *Solea senegalensis* the existence of a Δ4 desaturase, enabling the biosynthesis of docosahexaenoic acid (DHA) from eicosapentaenoic acid (EPA), which can be modulated by the diet. The present study aims to evaluate the combined effects of the partial replacement of fish oil (FO) with vegetable oils and reduced environmental salinity in the fatty acid composition of relevant body compartments (muscle, hepatocytes and enterocytes), the enzymatic activity over α-linolenic acid (ALA) to form n-3 LC-PUFA through the incubation of isolated hepatocytes and enterocytes with [1-^14^C] 18:3 n-3, and the regulation of the *S. senegalensis fads2* and *elovl5* in the liver and intestine. The presence of radiolabelled products, including 18:4n-3, 20:4n-3 and EPA, provided compelling evidence that a complete pathway enabling the biosynthesis of EPA from ALA, establishing *S. senegalensis*, has at least one Fads2 with ∆6 activity. Dietary composition prevailed over salinity in regulating the expression of *fads2*, while salinity did so over dietary composition for *elovl5*. FO replacement enhanced the proportion of DHA in *S. senegalensis* muscle and the combination with 20 ppt salinity increased the amount of n-3 LC-PUFA in hepatocytes.

## 1. Introduction

Traditionally, the oceans are the main source of omega-3 (or n-3) long-chain (≥C_20_) polyunsaturated fatty acids (LC-PUFA) because macroalgae, microorganisms and some invertebrates have the necessary enzyme repertoire for their de novo synthesis [1,2,3,4,5]. Importantly, n-3 LC-PUFA, such as eicosapentaenoic acid (EPA, 20:5n-3) and docosahexaenoic acid (DHA, 22:6n-3), are transferred from the bottom of the marine food web up to organisms occupying higher trophic levels, including fish [4,6]. Indeed, the relation among the high content of DHA in the ocean food webs and the start of fish consumption has been discussed as a crucial step in the brain evolution in hominids [7,8]. Because many fisheries are currently overexploited or being exploited at their maximum sustainable limit [9,10], aquaculture is considered a promising candidate for meeting the increasing demand for fish (from 9.0 kg per capita in 1961 to 20.5 kg in 2018) and seafood products, currently accounting for approximately 50% of the seafood consumed worldwide [9]. Despite its remarkable expansion over the last decades, aquaculture is facing several challenges related to its sustainability. One particularly relevant aspect is the extensive use of fishmeal and fish oil (FO) in aquafeed formulations, with aquaculture being regarded to consume approximately 75% of the global production of these finite resources. Fishmeal and FO, derived to a large extent from capture fisheries, are being replaced by alternative ingredients, alleviating pressure on wild-fish stocks [11]. In this sense, vegetable oils (VO) are now commonly used ingredients in aquafeed formulations since, along with being readily available and inexpensive compared to FO, they are highly digestible and excellent sources of dietary energy [12,13,14]. However, VO are devoid of LC-PUFA such as EPA and DHA, healthy compounds for vertebrates present in FO and, to a lesser extent, in fishmeal. Consequently, high inclusion levels of VO in aquafeeds are often associated with a reduction in the nutritional value of fish-farming products for the human consumer, and can also compromise fish health and well-being in species with a low capacity to bioconvert C_18_ polyunsaturated fatty acids (PUFA), abundant in VO, into physiologically important LC-PUFA [14,15].

The extent to which dietary FO can be replaced by VO in diets for fish depends upon the requirements for essential LC-PUFA, including EPA, DHA and arachidonic acid (ARA, 20:4n-6), themselves varying according to developmental stage and fish species. Consequently, a complementary strategy to boost aquaculture sustainability consists of growing fish species with low dependence on the dietary input of pre-formed LC-PUFA and a high capacity to utilise VO to satisfy their physiological demands for LC-PUFA via endogenous production (biosynthesis) while ensuring a high nutritional value (i.e., rich in n-3 LC-PUFA) for consumers. The biosynthesis of LC-PUFA from the C_18_ PUFA precursors α-linolenic acid (ALA, 18:3n-3) and linoleic acid (LA, 18:2n-6) in vertebrates, including fish, is determined by the complement and function of two types of enzymes, namely fatty acyl desaturases (Fads) and elongation of very long-chain fatty acid (Elovl) proteins. Fads catalyse the insertion of double bonds (unsaturations) at a specific position between an existing one and the carboxyl group; they are commonly termed “Δx desaturases”, where “x” denotes the carbon with the new double bond counting from the carboxyl group [16]. With few exceptions [17], teleosts have *fads2* as the sole *fads*-like gene in their genomes. However, a number of *fads2* genes vary among species, ranging from none (e.g., Japanese pufferfish, *Takifugu rubripes*) to four (e.g., Atlantic salmon, *Salmo salar*). Importantly, functions of teleost Fads2 are highly diversified, including mostly enzymes with Δ6/Δ8 desaturase activity, but also Δ5 and Δ4 activities [16]. On the other hand, Elovl are enzymes that catalyse the first and rate-limiting condensation reaction in the fatty acid (FA) elongation pathway [16]. There exist eight different Elovl present in vertebrates (Elovl1-8) [18], among which Elovl5 and Elovl2 have well-established roles in LC-PUFA biosynthesis since they are highly efficient in the elongation of PUFA substrates with chain lengths varying from 18 to 22 carbons [16].

The Senegalese sole (*Solea senegalensis* Kaup, 1858) is a common flatfish species in the Mediterranean and Southern Atlantic waters with an important market value and high growth performance [19]. *S. senegalensis* adapts well to captivity, presenting natural spawning through the control of temperature cycle, and high larval survival [20]. From its LC-PUFA biosynthetic capacity standpoint, *S. senegalensis* is a particularly interesting model since it possesses a Δ4 Fads2 that enables this species to biosynthesise DHA from docosapentaenoic acid (DPA, 22:5n-3) via the so-called “Δ4 pathway” [21]. Such a DHA biosynthesising capacity opens the possibility to grow this species with lower FO-inclusion diets without compromising fish health and product quality. Consistently, larvae fed diets containing reduced levels of DHA and EPA survive adequately [22,23]; additionally, dietary replacement of marine ingredients with terrestrial sources has shown good growth performance in *S. senegalensis* juveniles without affecting flesh nutritional value [24,25,26]. Interestingly, as reported in other fish species [27,28,29], the pathways of LC-PUFA biosynthesis can be regulated through diet in *S. senegalensis.* Morais et al. [30] observed upregulation of both Δ4 *fads2* and *elovl5* as well as increased enzymatic activity in Senegalese sole juveniles fed a diet replacing 75% of dietary FO with VO in comparison with control fish fed a FO diet. In addition to diet, environmental factors, such as salinity, can also modulate the activity of the LC-PUFA biosynthetic pathways in fish [31,32,33]. While the specific mechanisms involved are not yet fully understood [18], the modification of ambient salinity arises as a potential cost-effective strategy to enhance the LC-PUFA biosynthetic pathways in species such as *S. senegalensis* that are capable of acclimatising to different osmotic conditions [34,35]; this can be easily set up in recirculation aquaculture systems (RAS) [20]. 

The aim of the present study was to evaluate the combined effects of diet and environmental salinity in the regulation of LC-PUFA biosynthesis in *S. senegalensis* juveniles. More specifically, using a 2 × 2 factorial design, we investigated the effects of two diets with varying LC-PUFA contents (“FO” and “VO” denoting high and low dietary LC-PUFA, respectively) and two salinity conditions (35 and 20 ppt) in the FA composition of relevant body compartments (muscle, hepatocytes and enterocytes), the enzymatic activity over ALA to form n-3 LC-PUFA through the incubation of isolated hepatocytes and enterocytes with [1-^14^C] 18:3 n-3, and the regulation of the *S. senegalensis* Δ4 *fads2* and *elovl5* [21] in the liver and intestine.

## 2. Results

### 2.1. Fatty Acid Composition of Muscle, Hepatocytes and Enterocytes

Regardless of the treatment, the muscle of *S. senegalensis* contained between 28.63% and 30.18% of saturated fatty acids (SFA) with 16:0 (18.86–19.86%) being its main individual component (Table 1). Monounsaturated fatty acids (MUFA) significantly varied between dietary groups, FO (FO35: 31.87%; FO20: 32.08%) vs. VO (VO35: 29.99%; VO20: 29.12%), where oleic acid (OA, 18:1n-9) was the most abundant FA. Additionally, LA was the main n-6 PUFA followed by ARA, which was higher in both fish groups fed VO (VO35: 1.20%; VO20: 1.20%) than in those receiving FO (FO35: 0.88%; FO20: 0.86%). DHA was the major component within n-3 PUFA, presenting higher relative levels in VO-fed groups (VO35: 18.61%; VO20: 18.44%) than in FO-fed groups (FO35: 15.26%; FO20: 14.41%), while EPA showed the opposite trend (VO35: 3.19%; VO20: 3.31% vs. FO35: 3.78%; FO20: 3.89%, respectively).

SFA represented between 31.39% and 35.74% of total FA in hepatocytes, with 16:0 (20.25–22.22%) as its major component (Table 2). Moreover, total MUFA ranged from 36.20% to 41.23% mainly due to 18:1n-9, which was highest in VO treatments (VO35: 25.97%; VO20: 23.95%). Total n-6 PUFA was more abundant in VO- than in FO-fish, mostly accounted by 18:2n-6 (VO35: 9.06%; VO20: 10.88% vs. FO35: 6.35%; FO20: 6.00%). VO35-fish contained the lowest total n-3 PUFA of all experimental groups mainly due to reduced levels of both 22:5n-3 and 22:6n-3. Additionally, 20:5n-3 was higher in both FO treatments (FO35: 1.90%; FO20: 1.74% vs. VO35: 0.86%; VO20: 0.93%).

As shown in Table 3, SFA was the most abundant group of FA in enterocytes from *S. senegalensis* (30.97–39.04%), which was significantly influenced by the dietary treatment. Specifically, 16:0 was higher in FO than in VO treatments (FO, 23.55% and 21.69%; VO, 17.77% and 16.74%). By contrast, no differences existed in MUFA among the experimental treatments, where 18:1n-9 was the major component. Total n-6 PUFA was higher in VO groups than in the FO groups, especially 18:2n-6 (VO35: 10.57%; VO20: 13.14% vs. FO35: 6.12%; FO20: 5.13%). However, total n-3 PUFA remained constant regardless of the treatment, although 18:4n-3 was significantly higher in VO fish (*p* < 0.05).

### 2.2. Incorporation of Radioactivity into Cell Total Lipids and Bioconversion of [1-^14^C] 18:3 n-3

The incorporation rate of [1-^14^C] 18:3n-3 was higher in enterocytes (86.24 ± 13.64 to 153.68 ± 20.00 pmol mg protein^−1^ h^−1^) than in hepatocytes (45.95 ± 9.42 – 76.74 ± 8.68 pmoL mg protein^−1^ h^−1^), with most of the radioactivity recovered as unmodified substrate (96.24–98.54%) regardless of cellular type and experimental regime (Table 4). The bioconversion activity of [1-^14^C] 18:3n-3 was measurable in a variable number of fish per treatment. However, the hepatocytes mainly showed elongation activity, whereas enterocytes presented slightly higher bioconversion rates than hepatocytes as well as a higher variety of products derived from elongation/desaturation processes (Table 4). Fish fed the VO diet generally showed higher elongase/desaturase activities than those fed with FO.

The products obtained from hepatocytes and enterocytes incubated with [1-^14^C] 18:3n-3 are shown in Table 5. Irrespective of the treatment, the elongation of [1-^14^C] 18:3n-3 up to 22:3n-3 was the most frequent metabolic rate in both cell types ranging from 1.66% to 1.78% and from 1.30% to 1.52% of total radioactivity in hepatocytes and enterocytes, respectively. Additionally, biosynthesis of EPA and DHA was more common in enterocytes, especially in those fish receiving the VO diet.

### 2.3. Diet and Salinity Regulation of Gene Transcription

**Figure 1:**
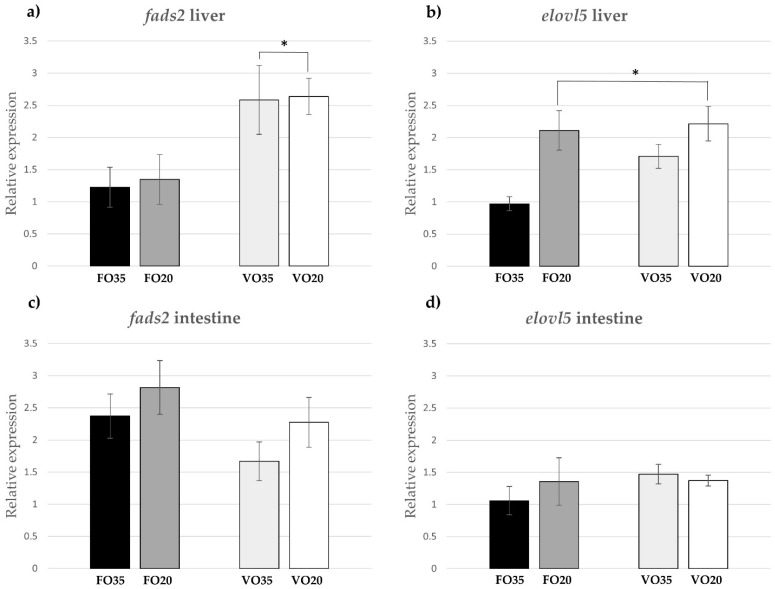
Distribution of *fads2* and *elovl5* mRNA levels in liver (**a**,**b**) and intestine (**c**,**d**) of *Solea senegalensis*. The relative expression is shown as geometric mean normalised expression ratios ± SE (*n* = 6). * Significant differences (*p* < 0.05).

Expression profiles of *fads2* and *elovl5* in the liver and intestine showed different patterns related to diet or salinity (Figure 1). In the liver, a higher expression of *fads2* was detected in both VO-fed fish groups (VO35 and VO20) compared to FO-fed fish (FO35 and FO20) (Figure 1a), while hepatic *elovl5* was upregulated in both 20 ppt treatments (FO20 and VO20) (Figure 1b). In contrast, the intestine presented no differences in the expression of either *fads2* or *elovl5*.

## 3. Discussion

The replacement of FO with alternative oils, such as VO, in aquafeeds has become an extended practice in the aquafeed manufacturing industry and it is largely perceived as a way to guarantee the sustainable intensification of finfish aquaculture [11,36,37,38]. Along with diet optimisation, the identification of species that are capable of utilising the plant-based diets that are already dominating the market might be a valuable strategy for supporting the sustainability of aquaculture. Within this context, *S. senegalensis* has been shown to have a higher LC-PUFA biosynthesising capacity compared to most of its marine counterparts [21,30]. In the present study, we investigated the combined effect of diet and salinity, the latter being regarded as an environmental factor through which LC-PUFA biosynthesis can be modulated in telosts [18,39].

The incubation of *S. senegalensis* hepatocytes and enterocytes with [1-^14^C] ALA demonstrated that EPA biosynthesis from ALA occurs in both cellular types, which is in agreement with previous studies suggesting that both tissues are active metabolic sites for LC-PUFA biosynthesis in fish [39]. While still poorly understood, the regulatory mechanisms operating in both tissues seem to differ as reported in Atlantic salmon *S. salar* [40] and gilthead seabream *Sparus aurata* [41]. Our assays indicated that both elongation and desaturation activities were slightly higher in enterocytes, confirming that, rather than regarded as a site where dietary lipids are simply absorbed and reacylated before being transferred elsewhere, the intestine plays an important function in LC-PUFA biosynthesis [42]. Our experiments also showed that elongation and desaturation of radiolabelled ALA in enterocytes were more common in fish fed the low LC-PUFA diet, especially when reared at 20 ppt, suggesting that the combination of both experimental conditions (low dietary LC-PUFA and low salinity) boosts the n-3 LC-PUFA biosynthesis from ALA. Interestingly, the presence of radiolabelled products, including 18:4n-3, 20:4n-3 and EPA, provided compelling evidence that a complete pathway enabling the biosynthesis of EPA from ALA is present in *S. senegalensis*. Thus, recovery of [1-^14^C] 18:4n-3 in enterocytes implies the existence of Δ6 desaturase activity mediating its biosynthesis from ALA. Subsequently, an Elovl5 can elongate 18:4n-3 to 20:4n-3, the latter being finally bioconverted into EPA by the action of a Δ5 desaturase. Results from our primary cell culture assays are thus in agreement with those of the functional assays of the *S. senegalensis* Elovl5 and Fads2 run in a heterologous expression system where transgenic yeast expressing the coding region of the *S. senegalensis* Elovl5 were able to elongate, among other C_18_ PUFA, 18:4n-3 into 20:4n-3 [21]. Moreover, the *S. senegalensis* Fads2 showed some capacity as Δ6 and Δ5 desaturase when expressed in yeast [21,43], and therefore arises as the candidate enzyme that accounts for the Δ6 and Δ5 desaturation capacities detected in our *S. senegalensis* cell preparations. It is worth noting that the *S. senegalensis* Fads2 is often regarded as a “Δ4 desaturase” since this was its most prominent capacity when assayed in yeast by Morais et al. [21], with % conversions above those for Δ6 and Δ5. While teleost Fads2 are often neo- and sub-functionalised enzymes [16], the multifunctionality of flatfish Fads2 is particularly remarkable. Indeed, the Δ6/Δ5/Δ4 desaturation abilities of the *S. senegalensis* Fads2 have also been reported in several flatfish species including *Trinectes maculatus*, *Apionichthys finis* and *Hypoclinemus mentalis* [44]. Interestingly, whereas the *T. maculatus* and *A. finis* Fads2 had Δ6 as their most prominent activity in yeast, the highest desaturase activity within the Δ6/Δ5/Δ4 Fads2 from *S. senegalensis* and *H. mentalis* was Δ4, raising the question of whether *S. senegalensis* possesses further *fads2*-like genes that could also contribute to the Δ6 and Δ5 desaturations observed in our in vitro assays. This is actually the case with *H. mentalis* that, along the high Δ4 trifunctional Fads2 alluded to above (termed “Fads2b” by Matsushita et al. [44]), has a second Fads2 ("Fads2a”) with ∆6 and ∆5 activities [44]. While the exact number of Fads2 in *S. senegalensis* remains to be elucidated, data collected from the present and other studies [21,43] allow us to establish that *S. senegalensis* has at least one Fads2 with ∆6 activity. This such presence has been recently discussed in the context of biosynthesis of the so-called “very long-chain (>C_24_) polyunsaturated fatty acids” by Elovl4 enzymes [45]. These authors suggested that production of 30:6n-3 and 32:6n-3, compounds identified in the eyes of *S. senegalensis* [46], occurs via consecutive elongation reactions catalysed by Elovl4 enzymes from 24:6n-3, a product of ∆6 desaturation from 24:5n-3 [45]. The expression of LC-PUFA biosynthesising genes, such as *fads2* and *elov5*, as well as the FA composition of tissues and cells analysed here indicate that a combined action of diet and salinity are effective ways to modulate LC-PUFA biosynthesis in *S. senegalensis* juveniles.

The FA composition of hepatocytes exhibited significant differences related to the interaction of salinity in the low LC-PUFA (“VO”) treatments. Specifically, the levels of 18:4n-3, DPA and DHA increased when fish were reared at 20 ppt compared to 35 ppt, suggesting a higher biosynthetic activity for forming DHA. Previously, the salinity has been proven to be an influential environmental factor in the composition of FA in other fish species, such as grey mullet (*Mugil cephalus*) [47], which showed higher levels of EPA and ARA when reared under conditions of reduced salinity. Additionally, the expression of *fads2* in the liver of the rabbitfish (*Siganus canaliculatus*) and red sea bream (*Pagrus major*) was higher in fish reared at low (10–15 ppt) compared to high salinities (32–33 ppt) [33,48]. Consistently, our results show that the expression of *elovl5* in liver was higher at a lower salinity, while the expression of *fads2* was higher in the low LC-PUFA treatments, in agreement with Morais et al. [30]. Thus, diet prevailed over salinity in regulating the expression of *fads2* while salinity prevailed over diet for *elovl5* expression, suggesting that VO20 was the best combination to stimulate the biosynthesis of LC-PUFA according to the upregulation observed for both candidate genes. Contrarily, none of the factors assayed (dietary LC-PUFA and salinity) significantly influenced the expression of *fads2* or *elovl5* in the intestine. Morais et al. [30] also reported that the expression of these genes in the intestine of *S. senegalensis* post-larvae was less affected by the diet than in the liver.

The FA composition of muscle, reflecting not only the impact of LC-PUFA biosynthesis but also other lipid metabolic processes, such as deposition, transport and catabolism [39,49], indicated that *S. senegalensis* juveniles can, to some extent, compensate for the limited provision of dietary LC-PUFA. DHA was higher in the high LC-PUFA diet than in the low LC-PUFA diet, whereas the relative content of DHA was higher in the muscle of the fish fed on the low LC-PUFA diet. This result could be possibly explained through the DHA biosynthesis and/or selective retention of the dietary DHA, since this essential FA is usually deposited in tissues at higher levels than those in the diet [50]. Moreover, the percentage of ARA was significantly higher in VO fish, in accordance with previous results for *S. senegalensis* larvae [51], suggesting that an excess of the C_18_ n-6 PUFA in the VO diet can be partially used to biosynthesise its C_20_ counterpart, similar, as described previously, to forming EPA from [1-^14^C] ALA. In addition, 22:5n-6, derived from ARA is present in fish muscle regardless of the treatment, probably due to a Δ4 activity [21], especially considering that this C_22_ FA was absent in the diets. Consistently with our results, 22:5n-6 raised up when *S. senegalensis* was fed increasing ARA levels [52], indicating the possible biosynthesis of this FA from ARA. 

In conclusion, our study demonstrated that FO replacement prevailed over salinity regulating the hepatic *fads2* expression while salinity did so over diet in *elovl5* expression, suggesting that a partial substitution of FO by VO can be a sustainable farming strategy to obtain commercial *S. senegalensis* rich in DHA, and that the combination with a lower salinity for a short period prior to slaughter enhances n-3 LC-PUFA biosynthesis in hepatocytes. 

## 4. Materials and Methods

The experiment complied with the Guidelines of the European Union Council (2010/63/EU) and the Spanish Government (RD1201/2005; RD53/2013 and law 32/2007) for the use of laboratory animals. All experimental protocols were approved by the Ethical Committee of the IFAPA (Andalusian Institute of Agricultural and Fisheries Research and Training), located in Seville, Spain.

### 4.1. Dietary and Salinity Modulation

A total of 24 Senegalese sole juveniles with an initial body weight of 490.8 ± 15.3 g were distributed into four 100 L tanks (6 fish per tank) at the facilities of Centro IFAPA Agua del Pino (Huelva, Spain) for 9 weeks (October 2017 to December 2017). Fish from two tanks were fed with a commercial *S. senegalensis* diet (LE-6 Europa RG; Skretting) (diet “FO”) while those from the other two tanks received an experimental diet (“VO”) consisting of 25% commercial FO diet and 75% commercial tilapia diet (TI-5 Tilapia; Skretting). For manufacturing the diets, both the FO and VO were triturated and repelletised. Samples were taken for lipid and FA analysis (Table 6). Fish were fed twice daily at a rate of 3–5% of biomass. Each diet was tested at 35 ppt and 20 ppt (control and low salinity, respectively), resulting in a total of four experimental treatments, namely FO35, FO20, VO35 and VO20. The rearing conditions during the experimental period were an average temperature of 18.5 ± 0.4 °C, dissolved oxygen above 5.0 ± 0.2 ppt and natural photoperiod.

### 4.2. Tissue Collection

At the end of the experiment, fish were starved for 24 h prior to being sacrificed through anaesthetics overdose (immersion in > 1 mL L^−1^ 2-phenoxyethanol). The specimens were individually measured and weighed, and samples of muscle, liver and intestine were collected. A portion of each tissue (~200 mg wet weight) was used for lipid determinations, whereas enterocytes and hepatocytes were isolated for in vitro metabolism studies with [1-^14^C] 18:3n-3. Finally, a portion of the liver and intestine (~100 mg wet weight) was collected in RNAlater^®^ and stored for the first 24 h at 4 °C and then frozen at −20 °C until further analysis of gene expression.

### 4.3. Lipid Analysis

Total lipids (TL) were extracted from diets, muscle and isolated cells (hepatocytes and enterocytes) according to Folch et al. [53] with small modifications [54]. Briefly, cell preparations were dissolved in 2 mL of 0.88% KCl (*w/v*) and 8 mL of chloroform/methanol (2:1, *v/v*) containing 0.01% (*w/v*) butylated hydroxytoluene (BHT) as an antioxidant. For the lipid extraction from diets, a sample of finely ground pellets (~100 mg) was hydrated with 0.5 mL of distilled water, and after 30 min at 4 °C, 5 mL of chloroform–methanol (2:1, *v/v*) were added to the solution, which was homogenised using a Virtis rotor homogeniser (Virtishear, Virtis, Gardiner, New York, USA) and kept overnight under a nitrogen atmosphere to prevent oxidation. Subsequently, a further 5 mL of chloroform–methanol (2:1, *v/v*) was added and re-homogenised, previous to the addition of 2.0 mL of KCl (0.88%, *w/v*). For the lipid extraction of muscle, the tissue was directly homogenised in 10 mL chloroform/methanol (2:1, *v/v*) with 2.5 mL of KCl (0.88%, *w/v*). The mixture was vigorously shaken, centrifuged at 716 g for 5 min, and the organic solvent was collected, filtered, and evaporated under a stream of nitrogen. The whole process was developed under an ice-cold environment to prevent sample degradation. The lipid content was determined gravimetrically, the extracts were re-suspended in chloroform/methanol (2:1, *v/v*) with 0.01% (*w/v*) BHT and stored at −20°C under a nitrogen atmosphere until further analysis. 

Up to 1 mg of TL extract of diet, muscle, hepatocytes and enterocytes without radiolabelled fatty acid (control) were subjected to acid-catalysed transmethylation to obtain fatty acid methyl esters (FAME). FAME were purified by thin-layer chromatography (Macherey-Nagel, Düren, Germany), separated and quantified using a TRACE-GC Ultra gas chromatograph (Thermo Scientific, Milan, Italy) equipped with an on-column injection, a flame ionisation detector (FID) and a fused silica capillary column Supelcowax TM 10 (30 m × 0.32 mm ID, df 0.25 μm) (Supelco Inc., Bellefonte, PA, USA). Helium was used as the carrier gas at 1.5 mL min^−1^ constant flow, and temperature programming was from 50 to 150 °C at a rate of 40 °C min^−1^, then from 150 to 200 °C at 2 °C min^−1^, to 214 °C at 1 °C min^−1^ and, finally, to 230 °C at 40 °C min^-1^, which was maintained for 3 min. Individual FAME were identified by reference to authentic standards (Mix C4-C_24_ and PUFA No. 3 from menhaden oil, Supelco Inc.) and to a well-characterised cod roe oil. Further confirmation of identity was carried out by GC-MS (DSQ II, Thermo Scientific, Austin, TX, USA) when necessary. The results are expressed as percentage of total FA.

### 4.4. Isolation and Incubation of Cells with [1-^14^C] 18:3n-3

Hepatocytes and enterocytes were obtained as described by Rodríguez et al. [55]. Briefly, the intestine was cleaned from food and faeces and the liver was perfused through the hepatic portal vein with a solution of marine Ringer (116 mM NaCl, 6 mM KCl, 1 mM CaCl_2_, 1 mM MgSO_4_, 10 mM NaHCO_3_, 1 mM NaH_2_PO_4_, 10 mM K_2_SO_4_ and 10 mM HEPES, at pH 7.4). Tissues were chopped with Hanks Balanced Salt Solution (HBSS) (NaCl 1.75%, 9.69 mM HEPES, 1.73 mM NaHCO_3_) and incubated with collagenase at 10 mg mL^−1^ by gently shaking at 20 °C for 40 min. The resultant cell suspension was filtered through a 100 µm nylon mesh with HBSS containing 1% fatty-acid-free bovine serum albumin (FAF-BSA). Cells were collected by centrifugation at 716 g for 10 min, washed with HBSS and re-centrifuged for 7 min. The whole experiment was developed under a cold environment to avoid tissue degradation. After isolation, each cell preparation was incubated for 3 h with 0.20 µCi of [1-^14^C] 18:3n-3 with specific activity of 114.8 dpm pmol^−1^. A control group of each cell type without radiolabelled FA supplement was also maintained under the same experimental conditions. After incubation, the cell viability was assessed by using the trypan blue exclusion test (>90% in all cases). Samples were stored at −80 °C until analysis.

The protein content of cells was determined according to Lowry et al. [56] using FAF-BSA as the standard; TL was extracted as described in Section 4.3. A 100 µg-aliquot of TL from cells incubated with radiolabelled FA was used to determine radioactivity incorporated using a liquid scintillation β-counter (TRI-CARB 4810TR, Perkin Elmer, Jurong, Singapore). The results obtained in dpm were related to TL and protein contents and transformed to pmol mg protein^−1^ h^−1^.

To determine the elongation/desaturation of [1-^14^C] 18:3n-3, a 1 mg-aliquot of the TL extract from each incubated cell type was transmethylated by acid-catalysis and separated by argentation thin layer chromatography [55]. The TLC plates were developed in toluene/acetonitrile, where 50 µL of a standard with a mixture of radiolabelled FAs was loaded and put into closed Exposure Cassette-K (BioRad, Madrid, Spain) in contact with a radioactive-sensitive phosphorus screen (Image Screen-K, BioRad, Madrid, Spain) for two weeks. The screens were scanned by an image acquisition system (Molecular Imager FX, BioRad, Madrid, Spain) and the radioactivity of the FA substrates transformed into products was quantified by image analysis software (Quantity One ver. 4.5.2, BioRad, Madrid, Spain).

### 4.5. RNA Extraction

The total RNA was extracted following the RNA TRI Reagent (Sigma-Aldrich, Saint Louis, MO, USA) extraction protocol. Tissue samples (~100 mg) previously fixed in RNA later were homogenised in 1 mL TRI Reagent in 1.5 mL Eppendorf tubes using a Mini-Beadbeater (Bio Spec Products Inc., Bartlesville, OK, USA). Homogenised samples were incubated at room temperature for 5 min before they were centrifuged at 12,000× *g* for 10 min at 4 °C. The supernatants were then transferred into fresh Eppendorf tubes and 100 μL 1-bromo-3-chloropropane (BCP) was added. The tubes were then vigorously shaken by hand for 15 s, incubated at room temperature for 15 min, and centrifuged at 20,000× *g* for 15 min at 4 °C. The aqueous (upper) phase was transferred to fresh tubes and half the volume (per aqueous phase volume) of isopropanol and an RNA precipitation solution was added to precipitate the RNA. The mixtures were subsequently gently inverted six times, incubated for 10 min at room temperature and centrifuged at 20,000× *g* for 10 min at 4 °C. The RNA precipitate formed gel-like pellets on the bottom of the tubes. The supernatant was removed and the pellets were washed with 1 mL of 75% ethanol in ddH_2_O (*v/v*). The pellets were lifted from the bottom of the tube by flicking and inverting the tubes a few times so that the entire surface of the pellets was properly washed. The tubes were then centrifuged at 20,000× *g* for 5 min at room temperature and the ethanol was carefully removed and discarded. This step was repeated twice. The RNA pellets were air dried at room temperature until all visible traces of ethanol were gone. Subsequently, RNA pellets were resuspended in ddH_2_O. The concentration and quality of RNA were assessed spectrophotometrically using the NanoDrop^®^ (ND-1000 spectrophotometer, LabTech International, Uckfield, U.K.). The quality and integrity of the RNA samples were further assessed by electrophoresis on 1% agarose gel (*v/v*). The RNA solutions were stored at −70 °C for further analysis.

### 4.6. First Strand cDNA Synthesis

First strand complementary DNA (cDNA) was synthesised using the High-Capacity cDNA Reverse Transcription Kit (Applied Biosystems™, Foster City, CA, USA) following the manufacturer’s instructions. The reverse transcription kits and the RNA were thawed on ice. A total of 10 μL of RNA solution containing 1 μg RNA in ddH_2_O were prepared in 0.2 mL PCR tubes, heated in a Biometra thermocycler for 5 min at 75 °C to denature RNA and held at 12 °C. The cDNA reverse transcriptase master mix was prepared according to manufacturer’s instruction. A volume of 10 μL of the cDNA reverse transcriptase mix containing 2 μL of reverse transcriptase buffer, 0.8 μL dNTP mix, 0.5 μL Oligo dT, 1.5 μL reverse transcriptase random primers, 1 μL reverse transcriptase and 4.2 μL nuclease-free water was added to the 10 μL solution of denatured RNA, mixed gently and centrifuged briefly. These were then put in a thermocycler set at 25 °C for 10 min, 37 °C for 2 h, 85 °C for 5 min and 12 °C for 4 min, after which the resultant cDNA was stored at −20°C.

### 4.7. Real-time Quantitative PCR (qPCR)

Expression of the *S. senegalensis fads2* and *elovl5* genes was determined by quantitative real-time PCR (qPCR) in the liver and intestine. Replicates for treatment were n=6 for each tissue and gene. Elongation factor 1 α (*ef1α*), ribosomal protein S4 (*rps4*) and ubiquitin (*ubiq*) were used as reference genes to assess the expression of target genes. To determine the efficiency of the primer pairs by Morais et al. [21], serial dilutions of pooled cDNA were carried out. The qPCR was performed on a Biometra TOptical Thermocycler (Analytik Jena, Jena, Germany) in 96-well plates in duplicates at total volumes of 20 µL containing 10 µL of Luminaris Color HiGreen qPCR Master Mix (Thermo Scientific, Carlsbad, CA, USA), 1 µL of each primer (10 µM), 2 µL or 5 µL of cDNA (1/20 dilution) for reference and target genes respectively, as well as 6 or 3 µL of molecular biology grade water. Negative controls (NTC, no template control), containing 5 µL molecular biology grade water, instead of templates, were also run. The qPCR conditions included a first step of activation at 50 °C for 2 min, then 95 °C for 10 min followed by 35 cycles of the denaturation step at 95 °C for 15 s, the annealing temperature (Table 7) for 30 s and a final step of extension at 72 °C for 30 s. After amplification, a melt curve of 0.5 °C increments from 60 to 90 °C was performed to confirm a single product in each reaction. The relative expression of *fads2* and *elovl5* among treatments was calculated as arbitrary units after normalisation by dividing by the expression level of the geometric mean of the housekeeping genes (*ef1α*, *rps4* and *ubiq*). Arbitrary units were obtained for each target gene (*fads2* and *elovl5*) and tissue from the ratio between the expression level of each of them and the average of the control treatment (FO35).

### 4.8. Statistical Analysis

Prior to analysis, the FA composition and the relative expression of *fads2* and *elovl5* genes were examined for normal distribution by the Shapiro–Wilk test, and for homogeneity of the variances with the Levene test. When normality and/or homoscedasticity were not achieved, the arcsine square root or ln(x) transformation was carried out. Subsequently, two-way ANOVA was used to determine the combined effects of the factors diet (FO or VO) and salinity (35 or 20 ppt) and their interaction. Significant differences were established for *p* values lower than 0.05. All statistical analyses were carried out using the IBM SPSS statistics 25.0 for Windows (SPSS Inc., Armonk, NY, USA).

## Figures and Tables

**Table 1:** Total lipid (% wet weight), total fatty acids (mg fatty acid/100 g wet weight) and main fatty acid composition (% of total fatty acids) of muscle from *Solea senegalensis*.

	FO35	FO20	VO35	VO20	Two-Way ANOVA		
					Diet	Salinity	Interact.
*Total lipid*	2.10 ± 0.14	1.96 ± 0.05	1.31 ± 0.08	1.33 ± 0.04	*		
*Total FA*	1615.24 ± 143.08	1519.29 ± 64.83	953.42 ± 58.50	962.08 ± 51.94	*		
14:0	5.12 ± 0.45	5.16 ± 0.41	4.13 ± 0.23	4.17 ± 0.53	*		
16:0	19.22 ± 0.46	19.86 ± 0.81	19.20 ± 0.60	18.86 ± 0.21			
18:0	3.07 ± 0.33	3.11 ± 0.49	3.58 ± 0.17	3.85 ± 0.45			
∑ *SFA*	29.44 ± 0.59	30.18 ± 0.85	28.68 ± 0.60	28.63 ± 0.26			
16:1n-7	6.46 ± 0.49	6.63 ± 0.45	5.31 ± 0.18	5.25 ± 0.61	*		
18:1n-9	17.78 ± 0.50	18.26 ± 0.30	17.82 ± 0.75	17.49 ± 0.79			
18:1n-7	3.47 ± 0.10	3.52 ± 0.13	3.19 ± 0.09	3.11 ± 0.15	*		
20:1n-9	1.70 ± 0.10	1.68 ± 0.08	1.51 ± 0.09	1.48 ± 0.11			
∑ *MUFA*	31.87 ± 1.11	32.08 ± 0.80	29.99 ± 0.60	29.12 ± 1.70	*		
18:2n-6	6.25 ± 0.30	6.10 ± 0.40	6.72 ± 0.30	7.02 ± 0.33			
18:3n-6	nd	nd	nd	nd			
20:3n-6	nd	nd	nd	nd			
20:4n-6	0.88 ± 0.13	0.86 ± 0.13	1.20 ± 0.05	1.20 ± 0.22	*		
22:4n-6	nd	nd	nd	nd			
22:5n-6	0.30 ± 0.10	0.36 ± 0.04	0.32 ± 0.10	0.31 ± 0.10			
∑ *n-6 PUFA*	7.66 ± 0.22	7.64 ± 0.30	8.53 ± 0.34	8.80 ± 0.27	*		
18:3n-3	1.37 ± 0.11	1.38 ± 0.14	1.23 ± 0.05	1.36 ± 0.16			
18:4n-3	1.21 ± 0.09	1.23 ± 0.11	0.90 ± 0.04	0.99 ± 0.13	*		
20:4n-3	0.50 ± 0.02	0.53 ± 0.04	0.43 ± 0.01	0.45 ± 0.04	*		
20:5n-3	3.78 ± 0.17	3.89 ± 0.28	3.19 ± 0.13	3.31 ± 0.15	*		
22:5n-3	5.02 ± 0.18	4.87 ± 0.30	5.19 ± 0.30	5.12 ± 0.20			
22:6n-3	15.26 ± 1.58	14.41 ± 1.01	18.61 ± 0.90	18.44 ± 2.16	*		
∑ *n-3 PUFA*	27.45 ± 1.30	26.60 ± 0.95	29.76 ± 1.05	29.94 ± 1.67	*		
∑ *n-3 LC-PUFA*	24.87 ± 1.48	23.99 ± 1.01	27.63 ± 1.08	27.58 ± 1.92	*		
n-3/n-6	3.60 ± 0.21	3.51 ± 0.17	3.51 ± 0.16	3.43 ± 0.27			

Values are means ± SE (*n* = 6); nd, not detected. FA, fatty acid; SFA, saturated fatty acids; MUFA, monounsaturated fatty acids; PUFA, polyunsaturated fatty acids; LC-PUFA, long-chain polyunsaturated fatty acids. Totals include other minor components not shown. * Significant differences (*p* < 0.05).

**Table 2:** Total lipid (mg lipid/mg protein), total fatty acids (µg fatty acid/mg protein) and main fatty acid composition (% of total fatty acids) of hepatocytes from *Solea Senegalensis*.

	FO35	FO20	VO35	VO20	Two-Way ANOVA		
					Diet	Salinity	Interact.
*Total lipid*	0.96 ± 0.15	0.81 ± 0.15	0.82 ± 0.18	0.70 ± 0.05			
*Total FA*	433.18 ± 76.73	373.97 ± 69.00	351.16 ± 78.11	294.28 ± 20.46			
14:0	3.88 ± 0.35	4.07 ± 0.13	3.07 ± 0.28	3.02 ± 0.08	*		
16:0	21.61 ± 0.89	21.95 ± 1.21	22.22 ± 0.40	20.25 ± 0.61			
18:0	3.97 ± 0.45	4.81 ± 0.47	5.11 ± 0.34	5.08 ± 0.70			
∑ *SFA*	31.46 ± 1.17	33.22 ± 2.03	35.74 ± 2.08	31.39 ± 1.22			
16:1n-7	6.22 ± 0.32	6.13 ± 0.26	5.03 ± 0.54	5.40 ± 0.25	*		
18:1n-9	20.08 ± 0.55	19.46 ± 0.70	25.97 ± 0.71	23.95 ± 0.65	*		
18:1n-7	4.81 ± 0.14	5.02 ± 0.27	5.27 ± 0.31	4.50 ± 0.14			*
20:1n-9	1.33 ± 0.03	1.24 ± 0.09	0.88 ± 0.17	nd	*		
∑ *MUFA*	36.37 ± 0.92	36.20 ± 1.04	41.23 ± 0.84	37.14 ± 0.90	*	*	
18:2n-6	6.35 ± 0.38	6.00 ± 0.26	9.06 ± 1.18	10.88 ± 0.33	*		
18:3n-6	nd	nd	nd	nd			
20:3n-6	nd	nd	nd	nd			
20:4n-6	0.82 ± 0.15	0.92 ± 0.12	0.62 ± 0.10	0.80 ± 0.11			
22:4n-6	nd	nd	nd	nd			
22:5n-6	nd	nd	nd	nd			
∑ *n-6 PUFA*	7.58 ± 0.41	7.28 ± 0.29	9.63 ± 1.28	12.25 ± 0.44	*		
18:3n-3	1.02 ± 0.08	0.92 ± 0.10	1.06 ± 0.27	1.43 ± 0.10			
18:4n-3	0.55 ± 0.05	0.63 ± 0.07	0.16 ± 0.07	0.38 ± 0.06	*	*	
20:4n-3	0.50 ± 0.04	0.32 ± 0.13	nd	nd	*		
20:5n-3	1.90 ± 0.34	1.74 ± 0.27	0.86 ± 0.23	0.93 ± 0.13	*		
22:5n-3	3.73 ± 0.68	3.10 ± 0.30	1.38 ± 0.13	2.61 ± 0.19	*		*
22:6n-3	13.39 ± 0.97	13.25 ± 2.14	6.08 ± 0.87	10.46 ± 0.63	*		*
∑ *n-3 PUFA*	21.15 ± 1.62	19.96 ± 2.56	9.91 ± 1.15	16.18 ± 0.63	*		*
∑ *n-3 LC-PUFA*	19.59 ± 1.57	18.40 ± 2.49	8.69 ± 0.93	14.37 ± 0.56	*		*
n-3/n-6	2.78 ± 0.11	2.72 ± 0.31	1.05 ± 0.06	1.33 ± 0.07	*		

Values are means ± SE (*n* = 5); nd, not detected. FA, fatty acid; SFA, saturated fatty acids; MUFA, monounsaturated fatty acids; PUFA, polyunsaturated fatty acids; LC-PUFA, long-chain polyunsaturated fatty acids. Totals include other minor components not shown. * Significant differences (*p* < 0.05).

**Table 3:** Total lipid (mg lipid/mg protein), total fatty acids (μg fatty acid/mg protein) and main fatty acid composition (% of total fatty acids) of enterocytes from *Solea senegalensis*.

	FO35	FO20	VO35	VO20	Two-Way ANOVA		
					Diet	Salinity	Interact.
*Total lipid*	1.21 ± 0.20	1.00 ± 0.27	0.32 ± 0.08	0.25 ± 0.04	*		
*Total FA*	341.54 ± 59.62	292.41 ± 71.56	85.15 ± 20.25	70.71 ± 12.43	*		
14:0	3.38 ± 0.32	3.22 ± 0.39	1.84 ± 0.19	1.62 ± 0.11	*		
16:0	23.55 ± 1.10	21.69 ± 1.76	17.77 ± 0.88	16.74 ± 0.44	*		
18:0	7.82 ± 0.44	8.46 ± 1.07	9.24 ± 0.43	8.45 ± 0.17			
∑ *SFA*	39.04 ± 1.39	36.81 ± 3.32	33.19 ± 0.81	30.97 ± 0.56	*		
16:1n-7	4.02 ± 0.55	4.00 ± 0.50	2.12 ± 0.23	2.39 ± 0.08	*		
18:1n-9	21.40 ± 1.54	20.49 ± 2.31	25.12 ± 3.26	23.78 ± 1.35			
18:1n-7	4.40 ± 0.21	4.21 ± 0.25	3.98 ± 0.22	4.15 ± 0.07			
20:1n-9	1.14 ± 0.08	1.31 ± 0.29	1.06 ± 0.35	1.04 ± 0.02			
∑ *MUFA*	33.71 ± 1.56	32.82 ± 3.36	34.78 ± 2.74	33.35 ± 1.63			
18:2n-6	6.12 ± 0.19	5.13 ± 0.42	10.57 ± 1.17	13.14 ± 1.42	*		*
18:3n-6	nd	nd	nd	nd			
20:3n-6	nd	nd	nd	nd			
20:4n-6	1.23 ± 0.16	1.64 ± 0.52	1.25 ± 0.17	1.42 ± 0.08			
22:4n-6	nd	nd	nd	nd			
22:5n-6	nd	nd	nd	nd			
∑ *n-6 PUFA*	7.64 ± 0.37	6.83 ± 0.96	12.06 ± 1.54	15.40 ± 1.63	*		
18:3n-3	1.05 ± 0.10	0.86 ± 0.04	0.89 ± 0.10	1.02 ± 0.03			
18:4n-3	0.50 ± 0.15	0.59 ± 0.04	1.28 ± 0.98	2.54 ± 0.47	*		
20:4n-3	nd	nd	nd	nd			
20:5n-3	0.64 ± 0.17	1.34 ± 0.31	0.54 ± 0.05	0.29 ± 0.16			
22:5n-3	2.39 ± 0.21	2.98 ± 1.14	2.20 ± 0.49	2.14 ± 0.13			
22:6n-3	9.93 ± 0.99	12.57 ± 4.02	10.21 ± 1.07	9.88 ± 1.19			
∑ *n-3 PUFA*	14.52 ± 1.24	18.34 ± 5.42	15.23 ± 1.49	15.97 ± 1.57			
∑ *n-3 LC-PUFA*	12.97 ± 1.33	16.90 ± 5.45	13.06 ± 1.60	12.42 ± 1.49			
n-3/n-6	1.89 ± 0.10	2.47 ± 0.42	1.27 ± 0.04	1.04 ± 0.09	*		

Values are means ± SE (*n* = 5); nd, not detected. FA, fatty acid; SFA, saturated fatty acids; MUFA, monounsaturated fatty acids; PUFA, polyunsaturated fatty acids; LC-PUFA, long-chain polyunsaturated fatty acids. Totals include other minor components not shown. * Significant differences (*p* < 0.05).

**Table 4:** Incorporation of radioactivity into total lipids (pmol mg prot^−1^ h^−1^) and bioconversions (% of total radioactivity) registered in isolated hepatocytes and enterocytes from *Solea senegalensis* incubated with [1-^14^ C] 18:3n-3.

Hepatocytes	FO35		FO20		VO35		VO20	
Incorporation	46.40 ± 4.31		45.95 ± 9.42		59.86 ± 17.46		76.74 ± 8.68	
FA recovery	98.41 ± 0.55	(5)	98.09 ± 0.84	(5)	98.01 ± 0.85	(5)	98.54 ± 0.44	(5)
Elongation	1.99 ± 0.49	(4)	2.26 ± 0.84	(4)	2.22 ± 0.63	(4)	1.83 ± 0.32	(4)
Desaturation	nd		nd		nd		nd	
E + D	nd		0.50	(1)	1.10	(1)	nd	
Unknown	nd		nd		nd		nd	
Enterocytes	FO35		FO20		VO35		VO20	
Incorporation	86.24 ± 13.64		108.84 ± 27.34		153.68 ± 20.00		131.27 ± 26.71	
FA recovery	97.75 ± 0.59	(5)	96.24 ± 1.77	(5)	97.67 ± 0.28	(5)	97.02 ± 0.70	(5)
Elongation	2.00 ± 0.41	(5)	1.95 ± 0.32	(4)	1.78 ± 0.54	(4)	2.25 ± 0.44	(5)
Desaturation	nd		1.99	(1)	nd		nd	
E + D	2.04	(1)	10.55	(1)	2.27 ± 0.30	(2)	0.92 ± 0.23	(4)
Unknown	nd		0.28	(1)	nd		nd	

Values are means ± SE. E + D, products which combine elongation and desaturation processes; nd, not detected. Values in brackets represent the number of fish with bioconversion detected.

**Table 5:** Products obtained (% of total radioactivity) from the incubation of isolated hepatocytes and enterocytes from *Solea senegalensis* with [1-^14^C] 18:3n-3.

Hepatocytes	FO35		FO20		VO35		VO20	
20:3n-3	nd		1.27	(1)	0.74	(1)	0.67	(1)
20:5n-3	nd		0.50	(1)	1.10	(1)	nd	
22:3n-3	1.78 ± 0.51	(4)	1.71 ± 0.25	(4)	1.76 ± 0.21	(4)	1.66 ± 0.29	(4)
24:3n-3	0.85	(1)	0.94	(1)	1.08	(1)	nd	
Enterocytes	FO35		FO20		VO35		VO20	
18:4n-3	nd		1.99	(1)	nd		nd	
20:3n-3	0.40 ± 0.07	(2)	0.26	(1)	0.57 ± 0.03	(2)	0.65 ± 0.21	(3)
20:4n-3	nd		2.84	(1)	nd		nd	
20:5n-3	2.04	(1)	2.81	(1)	2.27 ± 0.30	(2)	0.78 ± 0.28	(4)
22:3n-3	1.45 ± 0.26	(5)	1.44 ± 0.31	(4)	1.52 ± 0.34	(5)	1.30 ± 0.34	(5)
22:6n-3	nd		4.90	(1)	nd		0.28 ± 0.04	(2)
24:3n-3	0.66 ± 0.12	(3)	0.59 ± 0.29	(3)	0.46 ± 0.22	(2)	0.54 ± 0.15	(4)

Values are means ± SE. E + D, elongation and desaturation; nd, not detected. Values in brackets represent the number of fish with bioconversion detected.

**Table 6:** Proximate composition (% dry weight), total FA (mg fatty acid/g dry weight) and main fatty acid composition (% of total FA) of experimental diets.

	FO	VO
Ash (% DW)	11.04 ± 0.07	7.90 ± 0.09
Protein (% DW)	54.84 ± 0.90	45.55 ± 0.30
Total lipid (% DW)	17.06 ± 2.57	12.44 ± 0.24
Total FA (mg/g)	111.52 ± 9.03	98.56 ± 12.13
14:0	6.50 ± 0.18	3.46 ± 0.07
16:0	22.82 ± 0.73	18.22 ± 0.31
18:0	4.77 ± 0.10	4.45 ± 0.06
*∑ SFA*	37.41 ± 0.81	28.92 ± 0.37
16:1n-7	6.27 ± 0.22	4.24 ± 0.06
18:1n-9	14.64 ± 0.35	26.26 ± 0.32
18:1n-7	4.46 ± 0.20	4.55 ± 0.12
20:1n-9	2.12 ± 0.04	1.42 ± 0.00
*∑ MUFA*	29.46 ± 0.66	38.21 ± 0.51
18:2n-6	4.93 ± 0.04	14.89 ± 0.08
18:3n-6	nd	nd
20:3n-6	nd	nd
20:4n-6	0.65 ± 0.01	0.44 ± 0.02
22:4n-6	nd	nd
22:5n-6	nd	nd
*∑ n-6 PUFA*	5.58 ± 0.03	15.33 ± 0.10
18:3n-3	1.10 ± 0.02	2.63 ± 0.05
18:4n-3	1.47 ± 0.07	0.75 ± 0.04
20:4n-3	0.44 ± 0.03	0.13 ± 0.13
20:5n-3	7.47 ± 0.40	3.75 ± 0.21
22:5n-3	0.86 ± 0.06	0.46 ± 0.03
22:6n-3	8.85 ± 0.60	4.33 ± 0.27
*∑ n-3 PUFA*	20.19 ± 1.19	12.05 ± 0.72
*∑ n-3 LC-PUFA*	17.62 ± 1.10	8.67 ± 0.63
n-3/n-6	3.62 ± 0.23	0.79 ± 0.04

Values are means ± SE (*n* = 2); nd, not detected. FA, fatty acid; SFA, saturated fatty acids; MUFA, monounsaturated fatty acids; PUFA, polyunsaturated fatty acids; LC-PUFA, long-chain polyunsaturated fatty acids. Totals include other minor components not shown.

**Table 7:** Primers used for real-time quantitative PCR (qPCR) analysis of gene expression [21]. Shown are sequence and annealing temperature (T) of the primer pairs, size of the fragment produced, reaction efficiency and accession number of the target and reference genes.

Transcript	Primer Sequence (5′–3′)	Amplicon Size	T	Efficiency	Accession Number
*fads2*	AAGCCTCTGCTGATTGGAGA GGCTGAGCTTGAAACAGACC	131 bp	60 °C	0.92	JN673546
*elovl5*	TTTCATGTTTTTGCACACTGCGACACCTTTAGGCTCGGTTTT	161 bp	60 °C	0.91	JN793448
*Ubq*	AGCTGGCCCAGAAATATAACTGCGACA ACTTCTTCTTGCGGCAGTTGACAGCAC	93 bp	70 °C	0.95	AB291588
*rps4*	GTGAAGAAGCTCCTTGTCGGCACCA AGGGGGTCGGGGTAGCGGATG	83 bp	70 °C	0.96	AB291557
*ef1* *α*	GATTGACCGTCGTTCTGGCAAGAAGC GGCAAAGCGACCAAGGGGAGCAT	142 bp	70 °C	0.98	AB326302

## Data Availability

The data presented in this study are included in the corresponding sections throughout the manuscript.
